# A descriptive cross-sectional study of cholera at Kakuma and Kalobeyei refugee camps, Kenya in 2018

**DOI:** 10.11604/pamj.2020.37.197.24798

**Published:** 2020-10-29

**Authors:** Nereah Kisera, Christine Luxemburger, Nadia Tornieporth, George Otieno, Javan Inda

**Affiliations:** 1 Kenya Medical Research Institute (KEMRI) and Centres for Disease Control and Prevention (CDC), Nairobi, Kenya,; 2 Sanofi Pasteur Vaccine Epidemiology and Modelling, Lyon, France,; 3 University of Applied Sciences and Arts, Hanover, Germany,; 4 Turkana County Health Department, Turkana, Kenya

**Keywords:** Cholera, outbreak, vaccine, WaSH, refugees, Kakuma, Kalobeyei

## Abstract

**Introduction:**

cholera is a significant public health concern among displaced populations. Oral cholera vaccines are safe and can effectively be used as an adjunct to prevent cholera in settings with limited access to water and sanitation. Results from this study can inform future consideration for cholera vaccination at Kakuma and Kalobeyei.

**Methods:**

a descriptive cross-sectional study of cholera cases at Kakuma refugee camp and Kalobeyei integrated settlement was carried out between May 2017 to May 2018 (one year). Data were extracted from the medical records and line lists at the cholera treatment centres.

**Results:**

the results found 125 clinically suspected and confirmed cholera cases and one related death (CFR 0.8%). The cumulative incidence of all cases was 0.67 (95% CI=0.56-0.80) cases/1000 persons. Incidence of cholera was higher in children under the age of five 0.94(95% CI=0.63-1.36) cases/1000 persons. Children aged <5 years showed 51% increased risk of cholera compared to those aged ≥5 years (RR=1.51; 95% CI=1.00-2.31, p=0.051). Individuals from the Democratic Republic of Congo had nearly 9-fold risk of reporting cholera (RR=8.62; 95% CI=2.55-37.11, p<0.001) while individuals from South Sudan reported 7 times risk of cholera case compared to those from Somalia (RR=7.39; 95% CI=2.78-27.73, p<0.001).

**Conclusion:**

in addition to the improvement of water, sanitation and hygiene (WaSH), vaccination could be implemented as a short-medium term measure of preventing cholera outbreaks. Age, country of origin and settlement independently predicted the risk of cholera

## Introduction

Kenya is home to Kakuma, Kalobeyei and Daadab refugee camps. Kakuma and Kalobeyei are located in Turkana County, north-western Kenya [1]. Turkana County is located in the arid and semi-arid lands (ASALs) of Kenya which experiences droughts almost every five years [2]. The camps hosts individuals from Sudan, Somalia, Burundi, Somalia, Ethiopia, The Democratic Republic of Congo, Rwanda, Burundi, Eritrea and Uganda [3]. Turkana County is the poorest county in Kenya with 79 in every 100 of its population living in poverty. The host community are mainly pastoralists. There is a close interaction between refugees and Kenyans living in Turkana County; locals often trade and live among the refugees and frequently seek medical care from the camp hospitals [4].

Kenya has reported several cholera outbreaks over the years [5]. They regularly affect counties or districts with the highest poverty indices, like Turkana County [6]. The first cholera outbreak on record at Kakuma refugee camp was in 2005. Four hundred and eighteen (418) people were treated and four persons died [7]. In 2009, another outbreak was reported at the camp with 224 cases and four deaths recorded. The outbreak was thought to have been caused by an influx of 12,000 new refugees from Daadab refugee camp [8]. In 2015 UNHCR battled a severe cholera outbreak that affected over 1000 individuals and killed ten people at Daadab refugee camp [9].

Cholera outbreaks were responsible for 76 deaths in 20 of 47 Kenyan counties between January 2017 and November 2017, representing a case fatality rate of 1.9% with cases clustered in Kakuma, Kalobeyei and Daadab refugee camps, as well as densely populated areas of Nairobi [6]. The outbreak at Kakuma and Kalobeyei occurred in the backdrop of ongoing cholera epidemics in war-ravaged Somalia and South Sudan [10,11]. Some of the displaced persons ended up in refugee camps in Uganda, Kenya, Ethiopia and The Democratic Republic of Congo (DRC). Within this period, relief agencies reported cholera outbreaks in all host countries except Ethiopia [12].

The WHO advocates for the investment in water, sanitation and hygiene (WaSH) to prevent and control outbreaks [13]. Water scarcity is prevalent in Kenya; approximately, only 56% of people have access to clean water [14]. Results of a multivariate analysis found that the risk of cholera in Kenya can be ascribed to open defecation, lack of access to clean water, inadequate number of health facilities and a high poverty index [15]. Previous cholera outbreaks at Kakuma were linked to poor sanitary practices at the camp. Handwashing, use of clean water containers and raising awareness on the importance of hygienic practices, especially among newcomers to the camp, were recommended as likely interventions to reduce the risk of cholera transmission at the camp [7,8].

Kakuma and Kalobeyei received an influx of refugees in April 2017. Kakuma alone received 2533 children from South Sudan [16]. Health authorities reported facing a myriad of challenges that hindered effective implementation of WaSH. Despite recent improvements such as the installation of 37,500 litre plastic tanks, upgrading of the water distribution pipeline at Kalobeyei settlement and rapid action by cholera response team, existing health and sanitary infrastructure was quickly overwhelmed [14,15]. The UNHCR reported overcrowding, communal preparation and consumption of cold food and unhygienic food practices. The breakdown of water supply tanks and lack of adequate boreholes led people to source water from temporary river beds and other untreated sources of water [17-19].

The continued reporting of cholera outbreaks on a large scale in places like Yemen and Zimbabwe [20,21], especially among displaced populations has led to the serious consideration of vaccination using the current safe and effective oral cholera vaccine (OCV) as an adjuvant cholera prevention and control measure, which can be implemented in the short-to-medium term [22]. Most studies have shown that mass vaccination using OCV is feasible and highly acceptable among the target population including in Kenya; which has yet to implement mass vaccination using OCV [23-25].

Before the introduction of mass cholera vaccines in a particular setting, a comprehensive risk assessment should be carried out to clearly outline seasonal periods of outbreaks, the incidence of disease, attack rate and the population most at risk of getting cholera. This information will help in the allocation of resources and determine which age group to vaccinate based on specific geographic region and period; in Zanzibar, for example, cholera vaccination sites were chosen based on an analysis of historical epidemiological data [26,27]. Epidemiological studies of cholera in refugee camps like Kakuma and Kalobeyei are essential for future OCV concern.

## Methods

**Study design:** we conducted a descriptive cross-sectional study of cholera cases at Kakuma refugee camp and Kalobeyei integrated settlement. Data were extracted from the medical records and line lists at the cholera treatment centres between May 2017 to May 2018.

**Figure 1:**
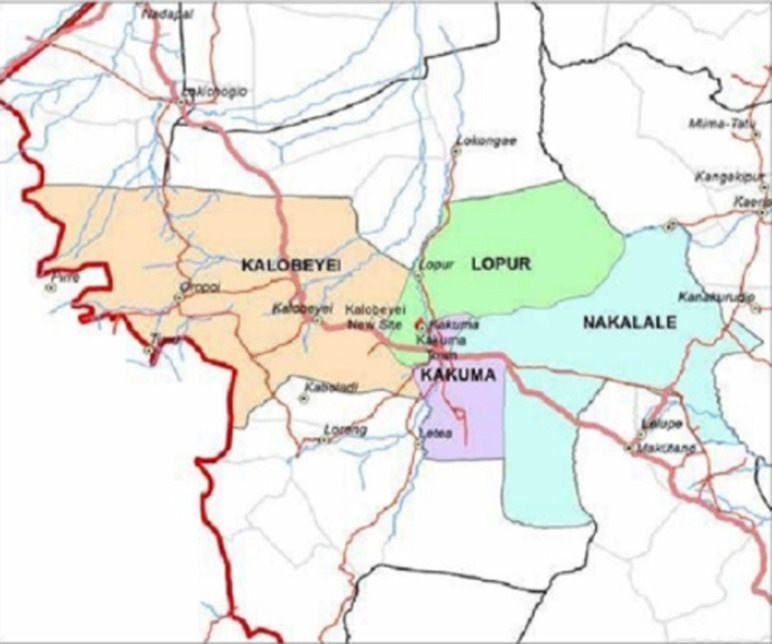
Kakuma and Kalobeyei regional location

**Study setting and population:** the outbreak occurred in two refugee camps situated in the arid and semi-arid lands of Turkana County, northern Kenya (Figure 1) [28]. Kakuma is a stable refugee camp established in 1992; following an influx of refugees in 2014 a separate settlement, Kalobeyei integrated settlement was established 25km away from Kakuma. Kakuma is divided into four camps, namely Kakuma I, Kakuma II, Kakuma III and Kakuma IV. Kalobeyei is administratively divided into village 1, 2 and 3 [1]. They host approximately 185,543 refugees and asylum seekers, mainly from neighbouring Eastern African countries [29].

**Cholera case definition:** a suspected case was defined as any patient who suffered from acute watery diarrhoea (at least three episodes in the last 24 hrs) and dehydration with or without vomiting. A case was confirmed by isolation of *Vibrio cholerae* from faecal samples. Once *V cholerae* was established from a few stool samples, the clinical case definition was sufficient to diagnose cholera cases. The definition of dehydration was based on the cholera outbreak response framework by the World Health Organization (WHO) [30].

**Laboratory testing:** stool samples were collected from cholera treatment centres using rectal swabs and transported to on-site laboratories using Cary-Blair medium. Rapid diagnostic tests were run on-site. Samples were taken to Kenya Medical Research Institute (KEMRI) laboratories for stool culture confirmation.

**Case detection and data collection:** all suspected cholera cases among refugees and the neighbouring community were referred to cholera treatment centres (CTC). We identified the cases by visiting the CTC and reviewing line lists and medical records. We collected retrospective clinical, demographic and epidemiological data using structured case report. Data was uploaded and fed directly to an excel database. Inclusion criterion was any person who fit the case definition of cholera and sought treatment at the cholera treatment centre and whose records could be found in patient records or line lists. Exclusion criterion was anyone who did not match the inclusion criteria.

**Statistical analysis:** we analysed data using STATA version 15 (StataCorp, College Station, TX). We used frequencies and percentages to describe the socio-demographic characteristics of the study population. Chi-square/Fisher´s exact test was used to test for association between categorical variables and clinical symptoms and period of hospital admission. Incidence risk was the total number of suspected cases divided by the mid-study period population. Case fatality ratio was calculated as the total number of cholera related deaths divided by the number of incidents multiplied by 100. The risk ratio was calculated for age group, sex, country of origin and settlement. All comparisons were made at a 95% confidence interval.

**Ethical consideration:** the study was undertaken with ethical approval from the Jaramogi Oginga Odinga Teaching and Referral Hospital Ethical Review Board. The research license was obtained from The National Commission for Science Technology and Innovation. Permission to visit the camp was received from the Ministry of Health, Kenya refugees' affairs secretariat; UNICEF branch office Nairobi, Kenya and the Turkana County government.

## Results

**Social-demographic characteristics of cholera cases:** we identified 125 clinically suspected cases of cholera of whom 107 were refugees and 18 non-refugees (Kenyans). One person died representing a case fatality rate (CFR) of 0.8% (Table 1). Rapid diagnostic tests were carried out on stool samples collected from 123/125 suspected cholera cases. Of those tested, 112 (89%) tested positive using RDTs. Out of the 30 stool samples transported to KEMRI, 22 (73%) were confirmed to be *V. cholerae* serotype, OI Inaba. The majority of the cases were aged ≥5 years (N=97; 78%). Individuals from South Sudan represented the majority of refugees settled in both camps. They made up more than 50% of the cases (N=86, 69%) recorded. The most heavily affected camp was Kalobeyei settlement with 51 (41%) cases reported (Table 1).

**Table 1:** sociodemographic characteristics of suspected cholera cases at Kakuma and Kalobeyei refugee camps, May 2017 to May 2018

	n	%
**Variable/factors**		
**Age (year)**		
<5	28	22
≥5	97	78
**Gender**		
Male	62	50
Female	63	50
**Country of origin**		
Burundi	4	3
DRC	11	9
Ethiopia	2	2
Kenya	18	14
Somalia	4	3
South Sudan	86	69
**Settlement**		
Kakuma	42	34
Kalobeyei	51	41
Other	32	26
**Camp number (Kakuma)**		
1	29	31.87
2	12	13.19
3	37	40.66
4	13	14.29

**Clinical symptoms:** all cases reported symptoms consistent with cholera. Among those aged ≥5 years, majority (N=87, 89.7%) manifested fever. Abdominal cramps were more prevalent among the under-fives compared to those aged ≥5 (75% vs 35%, p<0.001) (Table 2). Antibiotic sensitivity tests were carried out and patients were prescribed erythromycin, metronidazole and doxycycline. All cases reported exposure to untreated water at least one week before the onset of symptoms.

**Table 2:** clinical symptoms, outcomes and treatment data of suspected cholera cases at Kakuma refugee camp and Kalobeyei integrated settlement camp, May 2017 to May 2018

	Age group		
	<5 yrs (n=28)	≥5 yrs (n=97)	
	n (%)	n (%)	
**Fever (>37.5°C )**			
No	14 (50.0)	10 (10.3)	
Yes	14 (50.0)	87 (89.7)	
**Abdominal cramps**			**p <0.0001**
No	7 (25.0)	63 (65.0)	
Yes	21 (75.0)	34 (35.0)	
**Dehydration**			**p=0.317**
None	4 (14.3)	7 (7.2)	
Mild	21 (75.0)	70 (72.2)	
Severe	3 (10.7)	20 (20.6)	
**Period of hospital admission (days)**			**p=0.609**
0-1	5 (20.0)	23 (26.1)	
≥2	20 (80.0)	65 (73.9)	

**Incidence of suspected cholera cases at Kakuma and Kalobeyei refugee camp:** overall, the study reported cholera incidence of 0.67 per 1000 persons. Age, country of origin and settlement independently predicted the risk of cholera. Children ages below five years had a significantly higher incidence compared to those aged five years and above (0.94 per 1000 persons vs 0.62 per 1000 persons respectively). The risk of contracting cholera was about 51% higher among under-fives compared to those ages ≥5 (RR=1.51; 95% CI=1.00-2.31, p=0.051) (Table 3).

**Table 3:** incidence of suspected cholera cases at Kakuma and Kalobeyei refugee camp

	N	Mid period population	Incidence per 1000	Risk Ratio [95% CI]	p
**Overall**					
All cases	125	185543	0.67 [0.56-0.80]		
**Age (years)**					
<5	28 (22)	29695	0.94 [0.63-1.36]	1.51 [1.00-2.31]	0.051
≥5	97 (78)	155845	0.62 [0.50-0.76]	Ref.	
**Sex**					
Male	62 (50)	98864	0.63 [0.48-0.80]	Ref.	
Female	63 (50)	86859	0.72 [0.56-0.93]	0.86 [0.61-1.23]	0.415
**Country of origin**					
Burundi	4 (4)	10097	0.40 [0.11-1.01]	3.58 [0.67-19.20]	0.090
DRC	11 (10)	11524	0.95 [0.48-1.71]	8.62 [2.55-37.11]	<0.001
Ethiopia	2 (2)	10497	0.19 [0.02-0.69]	1.72 [0.16-12.00]	0.538
Somalia	4 (4)	36116	0.11 [0.03-0.28]	Ref.	
South Sudan	86 (80)	105091	0.82 [0.65-1.01]	7.39 [2.78-27.73]	<0.001
**Settlement/camp**					
Kalobeyei	51 (55)	38179	1.33 [0.99-1.76]	4.68 [3.11-7.04]	<0.001
Kakuma	42 (45)	147364	0.28 [0.21-0.39]	Ref	

* Fisher's exact P-value

In terms of country of origin, individuals from DRC and South Sudan reported the highest cholera incidence of 0.95 per 1000 persons and 0.82 per 1000 persons respectively. Despite having the second-largest population in the camp, individuals from Somalia reported the lowest incidence of 0.11 per 1000 persons. Individuals from DRC were nearly nine times more likely to report cholera cases compared to those from Somalia (RR=8.62, 95% CI 2.55-37.11), whereas individuals from South Sudan were seven times more likely to report cholera cases compared to those from Somalia (Table 3). The majority of cases among refugees living outside the camps occurred in refugee reception centres at Kakuma and Kalobeyei. Residents of Kalobeyei reported a higher incidence of cholera compared to Kakuma 1.33 per 1000 persons vs 0.28 per 1000 persons, with Kalobeyei resident reporting nearly 5-times risk of cholera cases compared to Kakuma (RR=4.68, 95% CI 3.11-7.04) (Table 3) even though a majority of the refugees who came in during an influx of April 2017 were reportedly settled at Kakuma which received 2533 children from South Sudan during this period.

## Discussion

Our analysis showed that children below five years of age Kakuma and Kalobeyei were at increased risk of infection compared to their adult counterparts. A study of cholera outbreaks between 1972-2010 in Kenya found that children below the age of 15 years make up for most of the cases in the country [31]. The disproportionate impact of cholera on children was also seen among Mozambican refugees in Malawi, where 60% of deaths reported were of children under the age of four [32]. This finding conforms to those of Mohammad *et al*. (2012), which found that children under the age of five are most adversely impacted by infection [33]. Our findings of the higher burden of cholera cases among under-five compared to the overall incidence are corroborated by the study by Deen *et al*. (2008) which reported the overall incidence versus the incidence under the age of five as; Jakarta (0.5/1000/yr vs 1.2/1000/yr), Kolkata (1.6/1000/yr vs 6.2/1000/yr) and Beira (4.0/1000/yr vs 8.8/1000/yr) [34]. Children, unlike adults, lack prior exposure to cholera antigens. They are more susceptible to infections as they cannot mount an anamnestic antibody response to infection; besides, children over the age of two years are more vulnerable to cholera due to waning maternal immunity [35,36].

Children have a higher tendency to develop complications post-infection. Dehydration is the most common complication in children [37]. Further evidence of the severity of cholera shows that loss of fluids and electrolytes is more significant in cholera compared to infection by rotavirus or *Escherichia coli* [37]. However, the results of the study did not show a significant difference in severity of dehydration between the under-fives and ≥5 populations. Cholera is an easily treatable and preventable disease. Effective management of cholera can reduce mortality in all ages to less than 1%. In refugee settings and internally displaced camps, the case fatality ratio can be higher than 5% [38]. Our study revealed a CFR 0.8% which was less than the national average (CFR, 1.9%) and lower than outbreaks in neighbouring countries like Somalia (CFR 6.6%) [39]. Improvement of WaSH infrastructure at the camps over the years and the rapid response by health authorities during the outbreak may have contributed to better outcomes [14,15] compared to previous cholera outbreaks which were linked to poor sanitary practices at the camp [7,8].

Our findings indicate that individuals from South Sudan and DRC were at increased risk of cholera while individuals from Somalia had the lowest risk of cholera compared to individuals from other countries. Those from South Sudan also accounted for 80% of the cholera cases during the 2005 cholera outbreak at Kakuma refugee camp [7]. This observed high incidence could be explained in parts by the fact that most refugees from South Sudan and DRC are new in the camp, hence there is a possibility that most of them had not developed immunity to cholera compared to their counterparts from Somalia who may have been either immune or asymptomatic cases. There was a sustained arrival of individuals from South Sudan and The DRC into Kakuma and Kalobeyei refugee camps in April 2017 which reportedly led to a breakdown of health and sanitation services and reliance on untreated water sources by camp residents in the resource strained camps [17]. Uganda, which hosts approximately 1 million South Sudanese refugees also reported outbreaks of cholera among newly arrived refugees from South Sudan and DRC Congo during the same period [11].

A number of the displaced people from Somalia are usually settled at Kakuma or Kalobeyei refugee camps. The majority of them end up at Daadab refugee camp located in North-Eastern Kenya [1]. In 2009, they accounted for the majority of the cholera cases (68%). During this time, Kakuma also received 12,000 new refugees from the Daadab refugee camp [8]. Previous studies did not ascertain the source of the outbreak, although, in one study, new arrivals at the camp were identified as a risk factor for infection [7]. The risk of cholera transmission by new arrivals was not determined during our analysis. Although our study did not investigate this, the low incidence of cholera among individuals from Somalia during this outbreak may be due to among other factors prior immunity from the 2009 outbreak. All the four camps at Kakuma are multi-cultural with the majority of residents being from South Sudan. There were marginally more cases reported from Kakuma camp 3. In a previous study, the attack rate at camp 2 (9.5 cases/1000 persons) was higher than the overall attack rate (2.7 cases/1,000) due to inequalities in the distribution of water and latrines [8].

The marginal difference between camps may have been due to an overall improvement in the delivery of sanitary services. The highest number of cases among those living outside the two camps was at the reception centres. During the time of the crisis, reception centres were reportedly overcrowded and unsanitary; conditions that would have put vulnerable refugees at risk of contracting cholera [17,18]. Kalobeyei refugee camp had five times the risk of cholera compared to Kakuma refugee camp. The reason for this could not be determined. However, a previous study pointed to disparities in the distribution of water and sanitation infrastructure between Kakuma and Kalobeyei [8]. The distinction between cases living in different villages at Kalobeyei could not be established during our analysis.

**Limitations:** the findings of the study may not be comprehensive as they relied on the data available inline lists and health records at the CTCs. The data for certain variables were at times missing, incomplete or inconsistent. This incidence of the outbreak may have also been higher than estimated as it only included symptomatic cases who presented at cholera treatment centres. Because of the limited variables in the line list, analysis of the impact of the outbreak on susceptible groups such as pregnant women, HIV positive individuals and the malnourished could not be done. Future studies may utilise more social demographic data to carry out an in-depth analysis of risk factors.

## Conclusion

Kakuma and Kalobeyei refugee camps still experience protracted outbreaks of cholera despite improvement in WaSH and better case management during outbreaks. Age, country of origin and settlement independently predicted the risk of cholera. The study findings may provide greater insight into the design of prevention and control strategies. In addition to the continued implementation of WaSH, vaccination measures with OCV could be implemented as a short-medium term measure of preventing future outbreaks taking into account age, country of origin and settlement area.

### What is known about this topic

- Sub-Saharan Africa has continuously recorded the highest number of cholera cases and case fatality ratios. Cholera infections most adversely impact children. Children under the age of five account for over half of the cholera deaths each year;
- Refugee camps are well-documented foci for cholera outbreaks;
- The mainstay for prevention of cholera outbreaks in refugee camps should be an investment in WaSH, health education, surveillance, cholera treatment with rapid rehydration, antibiotic treatment and the complementary use of OCV.

### What this study adds

- The real global burden of cholera is unknown. The number could be higher because of under-reporting of cases, particularly in sub-Saharan Africa. There have been very few studies describing cholera outbreaks at Kakuma and Kalobeyei refugee camps. Our study highlights the epidemiological burden of cholera which may provide greater insight into the design of prevention and control strategies;
- Our study suggested a higher risk of cholera among children below the age of five compared to the population above five years of age. Previous studies carried out at Kakuma and Kalobeyei did examine the risk of cholera disease among different age-groups. Our findings further highlight the continued vulnerability of children to cholera;
- The programmatic capacity to vaccinate many people and implementation of previous OCV campaigns depends on adequate surveillance data to ascertain the vulnerability and susceptibility of the affected group. Information on the epidemiological trends of cholera disease at the camps offers evidence for the use of cholera vaccines to prevent protracted outbreaks at Kakuma and Kalobeyei refugee camps.
